# Talking through Technology: Maintaining Essential Contacts with Older Adults During the Covid-19 Pandemic

**DOI:** 10.1093/geroni/igab046.2740

**Published:** 2021-12-17

**Authors:** Marcia Shade, Sarah Hubner, Julie Blaskewicz Boron, Stephen Kotopka, Natalie Manley

**Affiliations:** 1 University of Nebraska Medical Center, Omaha, Nebraska, United States; 2 University of Nebraska Omaha, Omaha, Nebraska, United States

## Abstract

Older adults are at increased risk for contracting COVID-19 and are more vulnerable to poor outcomes. Public health efforts to prevent spread of COVID-19 resulted in widespread social/physical distancing; this changed adults’ regular communication with their essential contacts, warranting development of solutions for socialization to reduce loneliness, bolstering quality of life. Essential contacts provide social/emotional/physical care for community-dwelling or institutionalized adults. This study aimed to explore how essential contacts of older adults utilize technology to maintain social connection in response to COVID-19. Participants (N=156) aged 55+ completed a Qualtrics questionnaire via Amazon Mechanical Turk; demographic, social contact, and technology use data were collected. Respondents (M Age=62.2±4.9) were generally female (72.4%), white (89.7%), and a contact for an institutionalized adult (59%). Data were analyzed descriptively with binary regressions. Results revealed that volumes of general [X2(4,N=156)=37.69,p<.001], in-person [X2(4,N=156=37.84,p<.001], and distanced [X2(4, N=156)=27.69,p<.001] social interaction were significantly associated with the older adult’s environment (community-dwelling vs. institutionalized). In-person conversation was significantly associated with environment [X2(1,N=156)=29.38,p=0.001], while other technology-based communications (e.g., video-chat) were not. In-person conversation was positively predicted by the contact being a physical caregiver (B=2.324,p<.001), while smartphone use was positively predicted by being a social contact (B=1.287,p<.05). Findings suggest that although technology was used by participants to communicate with their older adult contacts across groups, environment and caregiver/contact type significantly influenced communication. It may be that, throughout COVID-19, dyads have relied on familiar methods of socialization, or that there is lack of access to more sophisticated technologies for communication. This warrants future investigation.

